# Supplementary material for: Causal Relationship between Aging and Anorexia Nervosa: A White-Matter-Microstructure-Mediated Mendelian Randomization Analysis
Source: Biomedicines. 2024 Aug 16;12(8):1874. doi: 10.3390/biomedicines12081874 (PMC11351342; doi:10.3390/biomedicines12081874)
Supplement: Supplementary file 1 [file biomedicines-12-01874-s001.zip › Supplementary Figure/Figure S4/Singe SNP plot of MR analysis for TL to ubm-b-1626.pdf]

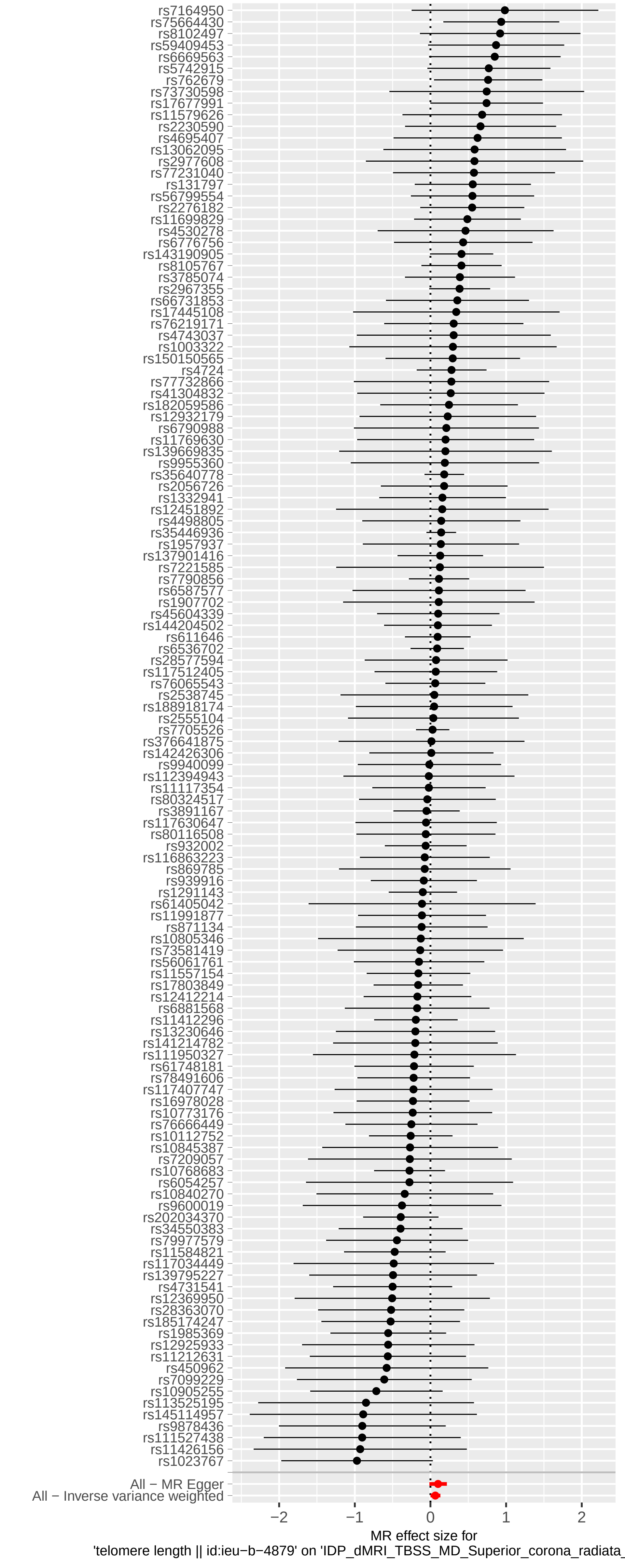
rs7164950
rs75664430
rs8102497
rs59409453
rs6669563
rs5742915
rs762679
rs73730598
rs17677991
rs11579626
rs2230590
rs4695407
rs13062095
rs2977608
rs77231040
rs131797
rs56799554
rs2276182
rs11699829
rs4530278
rs6776756
rs143190905
rs8105767
rs3785074
rs2967355
rs66731853
rs17445108
rs76219171
rs4743037
rs1003322
rs150150565
rs4724
rs77732866
rs41304832
rs182059586
rs12932179
rs6790988
rs11769630
rs139669835
rs9955360
rs35640778
rs2056726
rs1332941
rs12451892
rs4498805
rs35446936
rs1957937
rs137901416
rs7221585
rs7790856
rs6587577
rs1907702
rs45604339
rs144204502
rs611646
rs6536702
rs28577594
rs117512405
rs76065543
rs2538745
rs188918174
rs2555104
rs7705526
rs376641875
rs142426306
rs9940099
rs112394943
rs11117354
rs80324517
rs3891167
rs117630647
rs80116508
rs932002
rs116863223
rs869785
rs939916
rs1291143
rs61405042
rs11991877
rs871134
rs10805346
rs73581419
rs56061761
rs11557154
rs17803849
rs12412214
rs6881568
rs11412296
rs13230646
rs141214782
rs111950327
rs61748181
rs78491606
rs117407747
rs16978028
rs10773176
rs76666449
rs10112752
rs10845387
rs7209057
rs10768683
rs6054257
rs10840270
rs9600019
rs202034370
rs34550383
rs79977579
rs11584821
rs117034449
rs139795227
rs4731541
rs12369950
rs28363070
rs185174247
rs1985369
rs12925933
rs11212631
rs450962
rs7099229
rs10905255
rs113525195
rs145114957
rs9878436
rs111527438
rs11426156
rs1023767
All − MR Egger
All − Inverse variance weighted
−2
−1
0
1
2
MR effect size for
'telomere length || id:ieu−b−4879' on 'IDP_dMRI_TBSS_MD_Superior_corona_radiata_
